# Juvenile Arthritis Disease Activity Score (JADAS) based on CRP; validity and predictive ability in a Nordic population-based setting

**DOI:** 10.1186/1546-0096-9-S1-P155

**Published:** 2011-09-14

**Authors:** EB Nordal, M Zak, L Berntson, K Aalto, P Lahdenne, S Peltoniemi, S Nielsen, T Herlin, B Straume, A Fasth, M Rygg

**Affiliations:** 1 Department of Pediatrics, University Hospital of North Norway, Tromsø, Norway; 2 Department of Community Medicine, University of Tromsø, Norway; 3 Pediatric Rheumatology Department, Pediatric Clinic II, Copenhagen University Hospital, Rigshospitalet, Denmark; 4 Department of Women’s and Children’s Health, Uppsala University Hospital, Uppsala, Sweden; 5 Department of Pediatrics, Children’s Hospital, Helsinki University Hospital, Finland; 6 Faculty of Medicine, University of Helsinki, Finland; 7 Department of Pediatrics, Århus University Hospital, Denmark; 8 Department of Pediatrics, University of Gothenburg, Sweden; 9 Department of Laboratory Medicine, Children’s and Women’s Health, Norwegian University of Science and Technology and Department of Pediatrics, St.Olav’s Hospital, Trondheim, Norway. On behalf of the Nordic Study Group of Pediatric Rheumatology (NoSPeR

## Background

Juvenile Arthritis Disease Activity Score (JADAS) is a recently developed composite tool for scoring disease activity in juvenile idiopathic arthritis (JIA). JADAS consists of four items; the joint count, the physician and the patient’s/parent’s global assessment and the erythrocyte sedimentation rate (ESR) as an inflammatory marker. C-reactive protein (CRP) has been suggested as an alternative inflammatory marker [1].

## Aim

The aim of the study was to validate and compare the CRP versus the ESR as an inflammatory marker and to validate in JADAS in a Nordic population-based setting.

## Methods

We included newly diagnosed cases of JIA from defined geographical areas of Denmark, Finland, Sweden and Norway with disease onset in 1997 to 2000 followed longitudinally during the first eight years of the disease. The construct validity, predictive and discriminative ability, and sensitivity to change of JADAS were assessed by comparing with other measures of disease activity and outcome.

## Results

At the first study visit with all JADAS items available in 389 children, the correlation between JADAS27-CRP and JADAS27-ESR was 0.99. Children with higher JADAS scores had increased risk of concomitant pain, physical disability and use of disease modifying anti-rheumatic drugs (DMARD). Higher JADAS score at the first study visit also significantly predicted physical disability, damage, and DMARD during the disease course and no remission off medication at the final study visit. Median JADAS of the oligoarticular persistent category differed significantly compared to median score of all other categories (JADAS27-CRP; p=0.0002 and JADAS27-ESR; p=0.0004). Sensitivity to change was demonstrated as change in JADAS score compared to the American College of Rheumatology paediatric measures of improvement criteria, showed mostly excellent classification ability.

## Conclusions

The JADAS-CRP and JADAS-ESR correlate closely, show similar test characteristics, and are demonstrated to be feasible and valid tools to assess disease activity in all categories of JIA.
